# Impact of hydrogen gas inhalation during therapeutic hypothermia on cerebral hemodynamics and oxygenation in the asphyxiated piglet

**DOI:** 10.1038/s41598-023-28274-z

**Published:** 2023-01-28

**Authors:** Shinji Nakamura, Yasuhiro Nakao, Yinmon Htun, Tsutomu Mitsuie, Kosuke Koyano, Aya Morimoto, Yukihiko Konishi, Makoto Arioka, Sonoko Kondo, Ikuko Kato, Ken-ichi Ohta, Saneyuki Yasuda, Takanori Miki, Masaki Ueno, Takashi Kusaka

**Affiliations:** 1 Department of Pediatrics, Faculty of Medicine, Kagawa University, Mikicho 1750-1, Kitagun, Takamatsu, Kagawa 761-0793 Japan; 2 Medical Engineering Center, Kagawa University Hospital, Takamatsu, Kagawa Japan; 3 Maternal Perinatal Center, Faculty of Medicine, Kagawa University, Takamatsu, Kagawa Japan; 4 Department of Anatomy and Neurobiology, Faculty of Medicine, Kagawa University, Takamatsu, Kagawa Japan; 5 Postgraduate Clinical Education Center, Kagawa University Hospital, Takamatsu, Kagawa Japan; 6 Department of Pathology and Host Defense, Faculty of Medicine, Kagawa University, Takamatsu, Kagawa Japan

**Keywords:** Paediatric research, Translational research

## Abstract

We previously reported the neuroprotective potential of combined hydrogen (H_2_) gas ventilation therapy and therapeutic hypothermia (TH) by assessing the short-term neurological outcomes and histological findings of 5-day neonatal hypoxic-ischemic (HI) encephalopathy piglets. However, the effects of H_2_ gas on cerebral circulation and oxygen metabolism and on prognosis were unknown. Here, we used near-infrared time-resolved spectroscopy to compare combined H_2_ gas ventilation and TH with TH alone. Piglets were divided into three groups: HI insult with normothermia (NT, n = 10), HI insult with hypothermia (TH, 33.5 ± 0.5 °C, n = 8), and HI insult with hypothermia plus H_2_ ventilation (TH + H_2_, 2.1–2.7%, n = 8). H_2_ ventilation and TH were administered and the cerebral blood volume (CBV) and cerebral hemoglobin oxygen saturation (ScO_2_) were recorded for 24 h after the insult. CBV was significantly higher at 24 h after the insult in the TH + H_2_ group than in the other groups. ScO_2_ was significantly lower throughout the 24 h after the insult in the TH + H_2_ group than in the NT group. In conclusion, combined H_2_ gas ventilation and TH increased CBV and decreased ScO_2_, which may reflect elevated cerebral blood flow to meet greater oxygen demand for the surviving neurons, compared with TH alone.

## Introduction

Therapeutic hypothermia (TH) is the only standard treatment to minimize brain injury in hypoxic-ischemic (HI) encephalopathy (HIE) infants, achieving lower death and disability rates at 12–18 months^1–3^. However, this therapy does not prevent brain injury in all infants^2,4^. New agents that can augment the effects of TH are required to further improve outcomes.

Hydrogen (H_2_) gas became a major focus of research in neonatal medicine after the discovery of its potent antioxidative properties in vivo and in vitro for adult diseases such as cerebral ischemia^5–7^. H_2_ is considered an antioxidant, anti-inflammatory, and antiapoptotic agent that acts as a therapeutic and preventive antioxidant by selectively reducing the levels of highly active oxidants such as hydroxyl radical (•OH) and peroxynitrite (ONOO−) in cultured cells. As an adjunct to TH, we previously reported its neuroprotective potential through an assessment of the short-term neurological outcomes and histological findings of combined therapy in 5-day neonatal HIE piglets^8^. In particular, combined H_2_ gas ventilation and TH better ameliorated brain injuries compared with TH alone. However, neither the impact of H_2_ gas on cerebral hemodynamics and oxygenation nor its ability to improve prognosis are known.

Cerebral hemodynamics and oxygenation should be assessed in HIE neonates with or without TH because the changes in these parameters may be critical determinants of brain injury severity^9,10^. Three-wavelength near-infrared time-resolved spectroscopy (TRS) is an advanced near-infrared spectroscopy (NIRS) mode that can noninvasively and continuously measure not only cerebral hemoglobin oxygen saturation (ScO_2_) but also the absolute value of cerebral blood volume (CBV) at the bedside. Cerebral hemodynamics and metabolism can also be suppressed by successful treatment of asphyxia with TH^11^. However, we previously reported that piglets with severely suppressed neural activities after HI insult show further suppression of cerebral hemodynamics during TH after the insult, including a greater decrease in CBV, whereas piglets with severely suppressed neural activities show a greater increase in CBV during normothermia after the insult^10^. However, there are no reports on how H_2_ gas can impact cerebral hemodynamics and oxygenation during TH after HI insult.

We hypothesized that the combination of H_2_ gas ventilation with TH would alter cerebral hemodynamics and oxygenation after HI insult, thereby improving outcomes. Therefore, in this study, we compared the changes in CBV and ScO_2_ after HI insult in the piglet between combined H_2_ gas ventilation and TH and TH alone.

## Results

**Figure 1:**
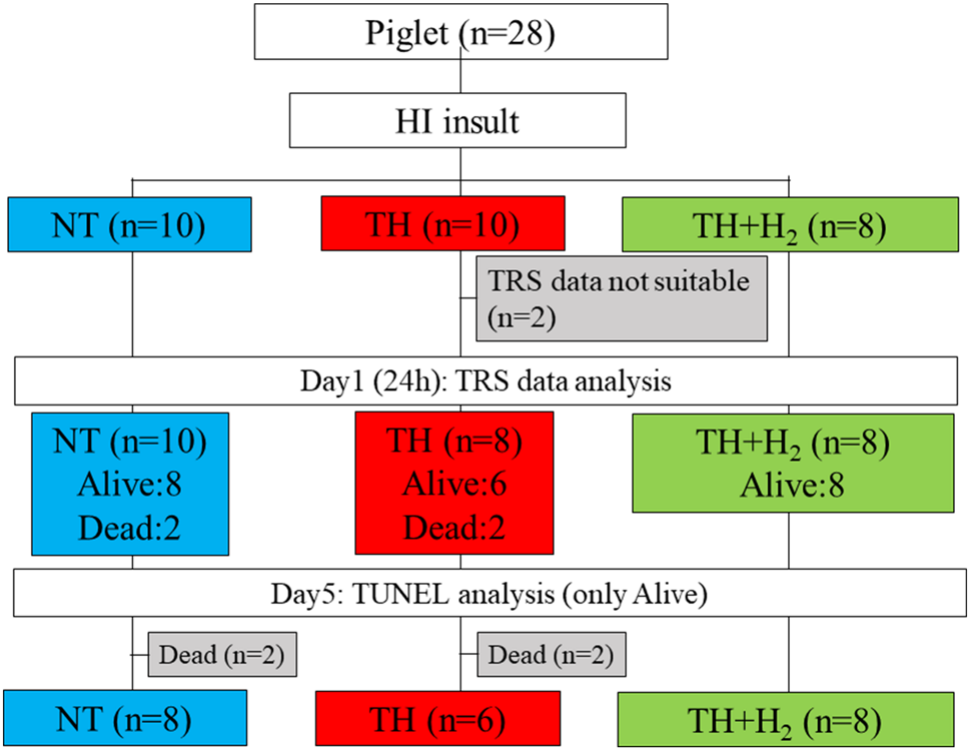
Study flow diagram.

The mean (SD) body weights were 1683 (189) g in the NT group [n = 10; five males (two died within 5 days after the insult) and five females], 1806 (11.5) g in the TH group [n = 8; eight males (two died) and two females], and 1804 (108) g in the TH + H_2_ group (n = 8; three males and five females). The four piglets died due to severe seizure. We excluded two piglets in the TH group because their ScO_2_ baseline values exceeded 80% and our previous reports showed that even under FiO2:1.0, the ScO_2_ value did not exceed 80% (Fig. 1). The duration of LAEEG after the insult did not differ among the groups [mean (SD): NT group, 25.4 (8.0) min; TH group, 26.2 (13.2) min; TH + H_2_ group, 25.4 (10.2) min].

### Histology

**Figure 2:**
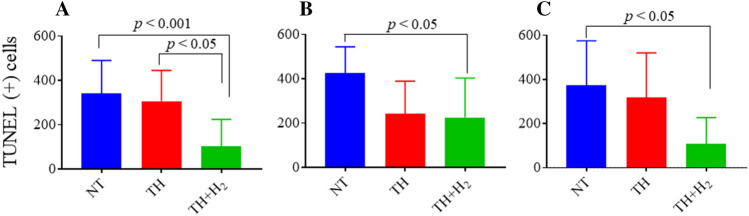
Number of TUNEL (+) cells in three regions of the cerebral cortex, namely, the dorsal cortex (**A**, DCx), sensorimotor cortex (**B**, SMCx), and mid-temporal cortex (**C**, MTCx). In the DCx, significantly fewer TUNEL (+) cells were seen in the TH + H_2_ group compared with the NT and TH groups (median with interquartile range). A *p* value < 0.05 was considered statistically significant. In the sensorimotor cortex (SMCx) and mid-temporal cortex (MTCx), the TH group showed significantly fewer cells compared with the NT group.

The histology results can be seen in our previous study^12^. Although there are slight differences from previous work in the numbers of newborn piglets and group compositions, these are largely the same results we reported previously. The TH + H_2_ group had significantly fewer TUNEL (+) cells in the dorsal cortex (DCx) compared with the NT and TH groups (Fig. 2A). The numbers of TUNEL (+) cells in the DCx were as follows: NT, 340.8 (259.8–452.8); TH, 306.3 (163.8–451.0); and TH + H_2_, 102.1 (0–137.5). In the sensorimotor cortex (SMCx; Fig. 2B) and mid-temporal cortex (MTCx; Fig. 2C), the TH group showed significantly fewer cells compared with the NT group. The values are expressed as median (interquartile range), and *p *< 0.05 was considered statistically significant.

### Arterial blood gas data and changes in physiological parameters after insult

Biochemical parameters such as PaO_2_, PaCO_2_, pH, base excess, lactate, glucose, and hemoglobin at baseline showed no significant differences among the three groups (Table 1). pH at 1 h after the insult was lowest in the TH group, while pH between 3 and 24 h after insult was lowest in TH + H_2_ group. PaCO_2_ was largely maintained at a constant value for 24 h after the insult in all groups and, PaO_2_ at 24 h after insult was highest in TH + H_2_ group. Hemoglobin was significantly lower 24 h after HI insult in the TH + H_2_ group than in the TH group.

**Table 1:** Blood gas, lactate, glucose, and hemoglobin before, at the end of insult, and at 1, 3, 6, 12, and 24 h after insult in the NT, TH, and TH + H_2_ groups.

	NT group	TH group	TH + H_2_ group
**pH**			
Baseline	7.42 (0.04)	7.40 (0.09)	7.45 (0.05)
0 h	6.83 (0.09)	6.79 (0.10)	6.92 (0.09)*^, ##^
1 h	7.30 (0.06)	7.19 (0.09)**	7.35 (0.07) ^####^
3 h	7.49 (0.04)	7.42 (0.08)**	7.41 (0.05)
6 h	7.48 (0.05)	7.44 (0.05)	7.41 (0.04)
12 h	7.47 (0.05)	7.46 (0.06)	7.38 (0.05)*
24 h	7.50 (0.06)	7.43 (0.04)	7.35 (0.05)****
**PaCO**_**2**_** (mmHg)**			
Baseline	46.4 (4.2)	45.9 (9.5)	41.9 (3.5)
0 h	36.2 (10.1)	41.3 (14.8)	39.4 (10.0)
1 h	43.0 (6.0)	45.0 (7.4)	38.8 (3.5)
3 h	43.1 (4.6)	40.1 (4.0)	45.2 (5.0)
6 h	45.8 (6.6)	43.5 (7.5)	44.2 (4.2)
12 h	44.3 (5.0)	41.1 (5.8)	44.5(3.1)
24 h	38.3 (4.4)	40.4 (7.0)	44.6(6.9)
**PaO**_**2**_** (mmHg)**			
Baseline	88.6 (11.2)	91.1 (13.0)	102.7 (5.5)
0 h	17.3 (6.0)	21.0 (6.0)	19.8 (5.5)
1 h	91.3 (24.8)	116.0 (22.1)*	122.4 (20.2)**
3 h	83.7 (18.4)	91.0 (26.1)	107.2 (12.5)
6 h	85.1 (14.2)	85.3 (23.1)	104.6 (17.5)
12 h	83.8 (14.5)	81.3 (26.3)	103.5 (17.4)
24 h	85.1 (19.5)	83.3 (1.6)	111.7 (26.1)*^, #^
**BE (mmol/L)**			
Baseline	5.7 (2.2)	2.8 (2.5)	4.5 (2.5)
0 h	− 26.0 (4.2)	− 27.3 (3.1)	− 22.1 (2.9)*^, ##^
1 h	− 5.1 (3.6)	− 10.6 (4.2)***	− 3.8 (3.7) ^###^
3 h	8.1 (1.6)	1.8 (3.6)***	3.5 (1.7)**
6 h	8.8 (1.4)	4.9 (2.6)*	3.0 (3.3)***
12 h	7.1 (3.0)	4.7 (1.6)	1.8 (3.1)**
24 h	6.0 (2.8)	2.3 (2.5)*	− 1.0 (3.8)****
**Lactate (mg/dL)**			
Baseline	16.3 (5.0)	20.3 (8.6)	17.6 (2.3)
0 h	225.4 (23.8)	210.9 (27.0)	175.1 (21.0)****^, ####^
1 h	119.2 (26.3)	118.9 (24.4)	93.9 (16.0)*^, ##^
3 h	33.3 (12.9)	55.9 (28.9)**	33.7 (7.9) ^##^
6 h	29.7 (9.2)	32.5 (13.2)	33.5 (6.8)
12 h	38.5 (10.5)	39.7 (13.8)	37.0 (6.1)
24 h	30.5 (11.7)	48.7 (10.7)	51.1 (23.0)
**Glucose (mg/dL)**			
Baseline	150.1 (18.4)	162.3 (28.1)	143.8 (18.8)
0 h	246.3 (63.1)	179.5 (98.6)	194.3 (83.9)
1 h	234.3 (50.3)	213.5 (82.6)	190.5 (51.1)
3 h	208.1 (58.6)	220.6 (68.9)	189.0 (50.0)
6 h	178.7 (49.2)	194.3 (62.7)	206.4 (53.9)
12 h	190.4 (65.2)	233.9 (75.4)	233.6 (36.5)
24 h	147.5 (73.1)	223.5 (81.5)*	187.1 (64.4)
**Hemoglobin (g/dL)**			
Baseline	10.1 (2.5)	9.7 (1.5)	9.3 (1.6)
0 h	10.7 (3.0)	10.3 (1.6)	10.3 (4.1)
1 h	10.4 (2.1)	10.8 (1.8)	9.1 (1.4)
3 h	10.8 (2.4)	11.5 (2.0)	10.4 (2.3)
6 h	10.9 (2.7)	12.4 (2.2)	10.7 (2.6)
12 h	10.7 (2.1)	12.2 (1.8)	11.1 (2.3)
24 h	9.8 (1.4)	11.9 (1.5)	8.8 (2.0)^#^

*p < 0.05, **p < 0.01, ***p < 0.001, ****p < 0.0001 versus NT, ^#^*p* < 0.05, ^##^*p* < 0.01, ^###^*p* < 0.001, ^####^*p* < 0.0001 versus TH by two-way ANOVA, post hoc Tukey analysis.

**Figure 3:**
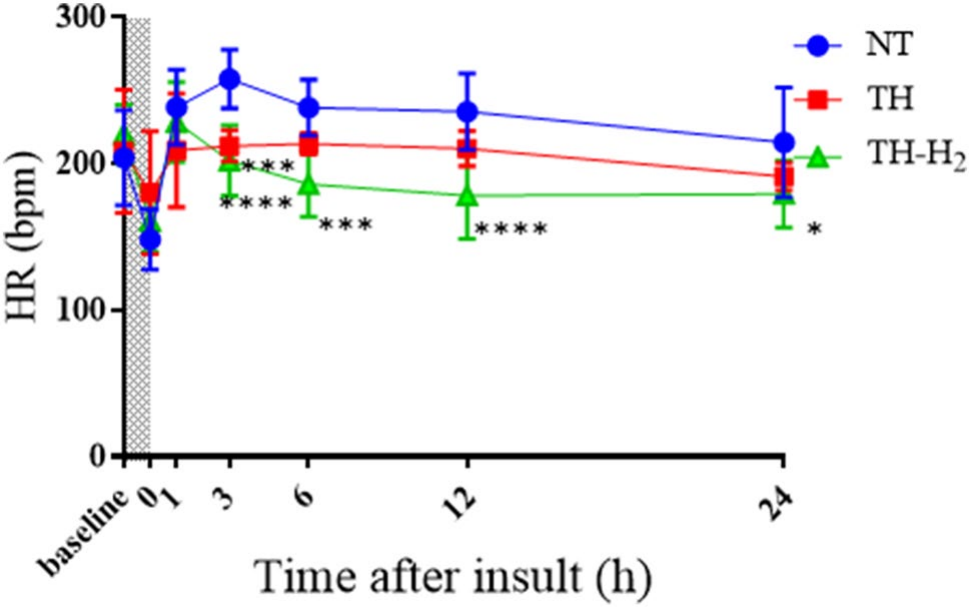
Heart rate (bpm) at the end and at 1, 3, 6, 12, and 24 h after hypoxic-ischemic insult in normothermia (NT, n = 10), therapeutic hypothermia (TH, n = 8), and TH with hydrogen gas inhalation (TH + H_2,_ n = 8) groups. Shaded areas indicate the hypoxic-ischemic insult period. Data are means ± SD in the NT group (blue circle), TH group (red square), and TH + H_2_ gas group (green triangle). ^*^p < 0.05, ^**^p < 0.01, ^***^p < 0.001, ^****^p < 0.0001 vs. the NT group.

**Figure 4:**
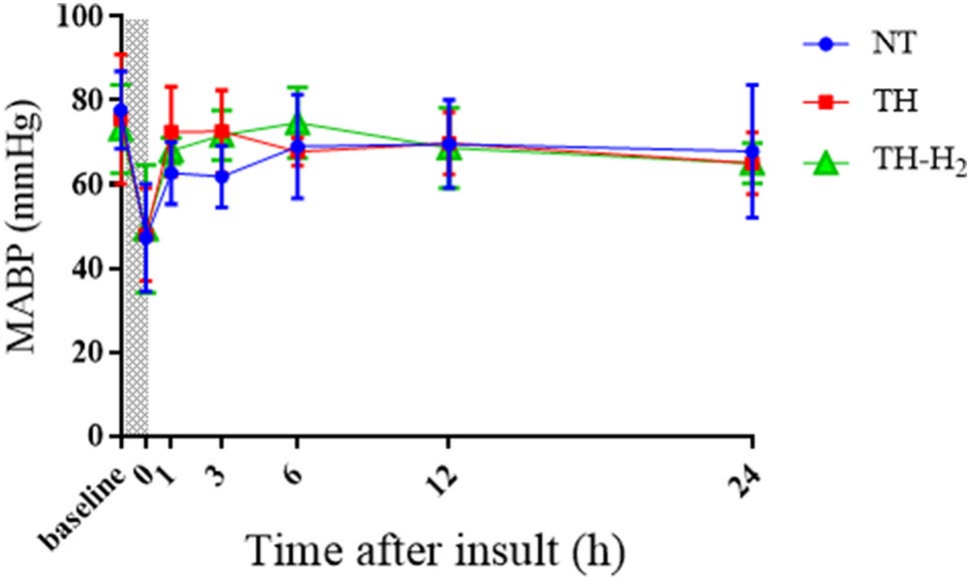
Mean blood pressure (MABP, mmHg) at the end and at 1, 3, 6, 12, and 24 h after hypoxic-ischemic insult in normothermia (NT, n = 10), therapeutic hypothermia (TH, n = 8), and TH with hydrogen gas inhalation (TH + H_2_, n = 8) groups.

Regarding HR after the initial resuscitation, all groups exhibited an immediate increase within 1 h after the insult. The NT group showed the highest HR compared with the other groups throughout the 24 h. In contrast, the TH + H_2_ group showed the lowest HR from 3 to 24 h of all groups (Fig. 3). For MABP, all groups exhibited increased MABP within 6 h after the insult, and from 12 h after the insult, MABP maintained a constant value in all groups (Fig. 4). There were no significant differences in MABP among the three groups throughout the overall experiment.

**Figure 5:**
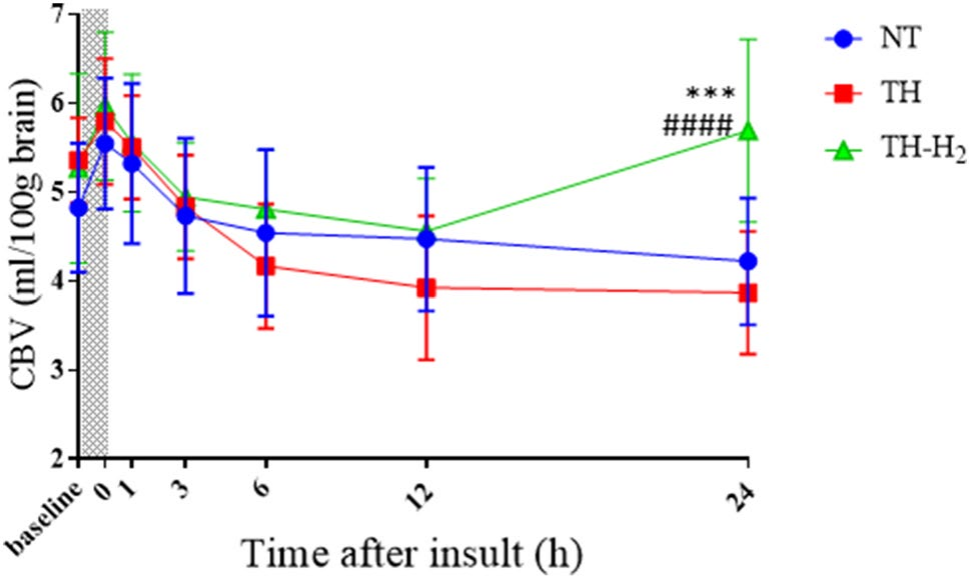
Cerebral blood volume (CBV, mL/100 g brain) at the end and at 1, 3, 6, 12, and 24 h after hypoxic-ischemic insult in normothermia (NT, n = 10), therapeutic hypothermia (TH, n = 8), and TH with hydrogen gas inhalation (TH + H_2_, n = 8) groups. ^***^p < 0.001 vs. the NT group; ^####^*p* < 0.0001 vs. the TH group.

**Figure 6:**
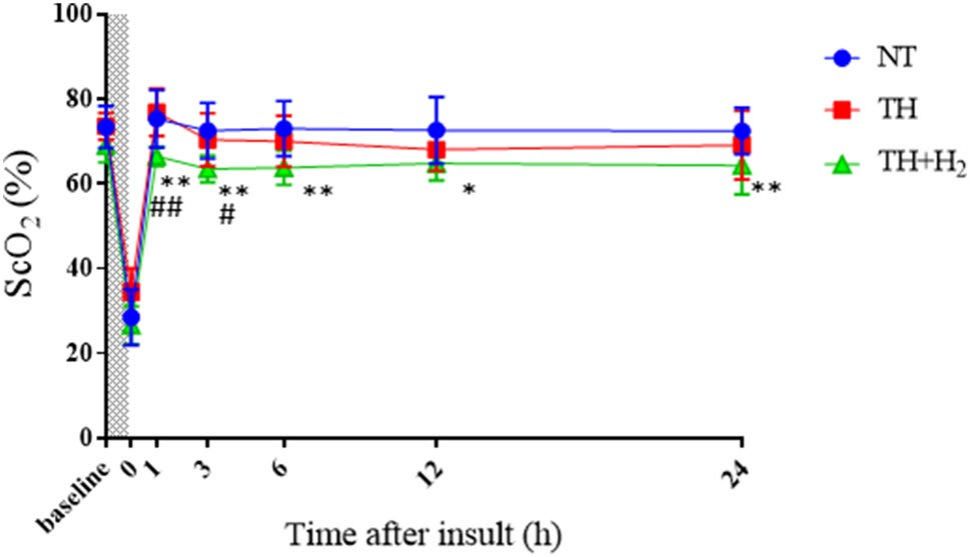
Cerebral hemoglobin oxygen saturation (ScO_2_, %) at the end and at 1, 3, 6, 12, and 24 h after hypoxic-ischemic insult in normothermia (NT, n = 10), therapeutic hypothermia (TH, n = 8), and TH with hydrogen gas inhalation (TH + H_2_, n = 8) groups. ^*^p < 0.05, ^**^p < 0.01 vs. the NT group; ^#^*p* < 0.05, ^##^*p* < 0.01 vs. the TH group.

For CBV and ScO_2_, all groups showed a decreased CBV until 12 h after the insult. At 24 h, the NT and TH groups still showed a decreased CBV, but it was significantly higher in the TH + H_2_ group (Fig. 5). In contrast, ScO_2_ was significantly lower in the TH + H_2_ group than in the NT group 24 h after the insult and was significantly lower at 1, 3 h compared with the TH group (Fig. 6).

### *Relationship between the change in CBV 24 h after HI insult and the number of TUNEL (*+*) cells*

**Figure 7:**
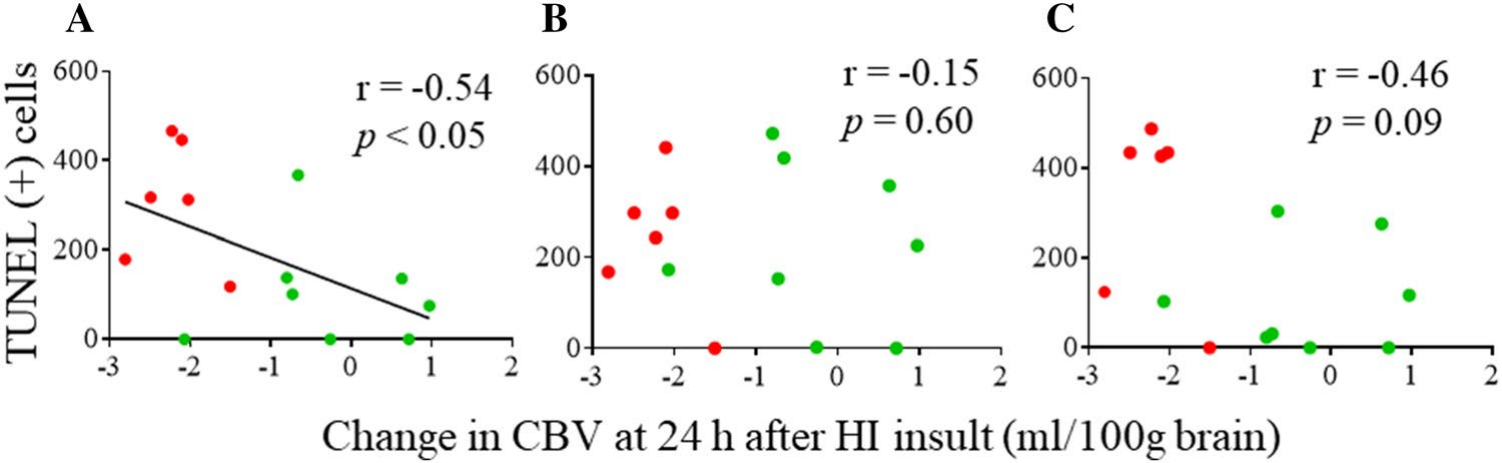
Relationship between the change in CBV 24 h after HI insult and the number of TUNEL (+) cells (**A**, DCx; **B**, SMCx; **C**, MTCx). The change in CBV 24 h after HI insult is calculated by subtracting the value at the end of the HI insult from the value 24 h after the HI insult. The change in CBV 24 h after HI insult showed a significant negative correlation with the number of TUNEL (+) cells in the DCx (A).

We examined the correlation between the change in CBV 24 h after HI insult (calculated by subtracting the value at the end of the HI insult from the value 24 h after the insult) and the number of TUNEL (+) cells in the cortex (Fig. 7) in the TH and TH + H_2_ groups. In Fig. 7A, the change in CBV 24 h after HI insult showed a significant negative correlation with the number of TUNEL (+) cells in the DCx.

## Discussion

In this study, we found that, compared with a TH group, our TH + H_2_ group showed (1) a lower HR with constant blood pressure, (2) a higher CBV and lower ScO_2_. These results indicate that combined H_2_ gas ventilation and TH might improve cerebral hemodynamics and oxygenation and thereby help to reduce brain injuries.

In previous animal studies, sheep and piglets with brain injuries showed increased cerebral blood flow (CBF) and CBV within 24 h after HI insult^13–15^. These cerebral hemodynamic changes might reflect a decrease in oxygen metabolism along with hyperemia due to secondary energy failure caused by impaired cerebral autoregulation. In the clinical setting, it has previously been reported that HIE neonates with adverse outcomes exhibited increases in CBV or ScO_2_ from 6 to 24 h after birth^9,16,17^. However, TH is likely to reduce CBF and CBV because it can induce a cooling-associated decrease in the cerebral metabolic rate^10,18^. Interestingly, it has been reported that fetal sheep with increased CBF during TH after HI insult had a better outcome^19^. In addition, we previously reported that a greater decrease in CBV during TH after an insult was correlated with more suppressed neural activities^10^.

In this study, we aimed to address how H_2_ gas inhalation alters cerebral circulation and oxygenation during TH. Using a neonatal pig model, we found that H_2_ gas in combination with TH was more effective than TH alone in reducing brain damage and accelerating motor function recovery. Nevertheless, the difference in cerebral circulation and oxygen metabolism changes between TH + H_2_ and TH alone had still been unclear.

We have previously reported the relationship of CBV and ScO_2_ with brain damage in the asphyxiated piglet, focusing on the use of TRS to measure cerebral circulation and oxygen metabolism. In those studies, we found that the relationship between CBV changes and brain damage was different for TH than for NT^10,14^. Thus, CBV was considered to be a useful indicator of the effect of treatment on cerebral circulation. We also hypothesized that the CBV changes in TH + H_2_ would be different from those in TH alone and that this difference in circulation might be related to the cerebroprotective effects of TH + H_2_ in the present study. Wintermark et al.^20^ found that, in human HIE infants with severe brain damage, as measured by MRI, high CBF and high ScO_2_ were also observed at the same time, and they speculated that this result may reflect a decreased oxygen demand despite an increased oxygen supply. In our study, in the TH + H_2_ group, we speculated that the higher CBV reflected higher CBF give that ScO_2_ was higher 24 h after HI insult and that ScO_2_ was low, not high, as a result of a greatly increased oxygen demand because normal cells were able to maintain their function. Although examination of the balance between cerebral oxygen demand and consumption is vital, this study was not able to analyze oxygen demand and oxygen consumption rates, and thus further investigation is needed.

Nevertheless, in clinical practice, it is not very feasible to transport a severely injured HIE infant to the MRI room for imaging. It is also difficult to continuously and noninvasively monitor CBF and CMRO_2_ at the bedside. Although TRS cannot measure these parameters, it can noninvasively and continuously measure not only ScO_2_ but also absolute CBV at the bedside at the same time, which is more useful for noninvasively and continuously estimating cerebral hemodynamics and oxygenation compared with ScO_2_ alone.

To our knowledge, this is the first study to show that H_2_ gas improves cerebral hemodynamics and oxygenation during TH after HI insult. TH is believed to protect against reperfusion injury via multiple mechanisms, including the suppression of free radicals, enzymes, and excitatory and inflammatory reactions^19,21^, in addition to the direct physical protection of membranes, similar to H_2_ gas^6^. However, unlike H_2_ gas, TH suppresses the cardiovascular system. We speculated that H_2_ gas may be able to improve cerebral and cardiovascular function under even TH conditions. Domoki et al. suggested that H_2_ ventilation increased cerebrovascular reactivity to hypercapnia after insult in the piglet^22^. In a rat model of global cerebral ischemia, inhalation of H_2_ gas palliated brain edema and blood–brain barrier disruption, reduced neuronal apoptosis, and improved neurological function^23^. Reactive oxygen species (ROS) directly destroy lipids, proteins, and nucleic acids, damaging vascular endothelial cells and the basement membrane^24^. Another study reported that H_2_ reduced hemorrhagic transformation in a focal cerebral ischemic/reperfusion rat model and that the reduction of oxidative agents might boost the survival of endothelial cells, neurons, and glial cells^25^. In the present study, we speculated that the reduction in potent ROS and the consequent decrease in brain edema may have been responsible for the increase in CBV and lower ScO_2_. Furthermore, Hayashida et al. reported that H_2_ gas inhalation can improve left ventricular function after the return of spontaneous circulation (ROSC) in the adult rat^26^, and their result showing a lower HR in the TH + H_2_ group compared with the TH group after ROSC is agreement with our results.

This study has some limitations. The mechanistic details of H_2_-induced neuroprotection remain unclear but likely involve inhibition of oxidative injury and neuroinflammation. However, we have virtually no information on the mechanism underlying the H_2_-induced neuroprotection observed in this study. Accordingly, we will examine biomarkers related to multiple mechanisms, including the suppression of free radicals, enzymes, and excitatory and inflammatory reactions, in addition to the direct physical protection of membranes. HI injury represents a complex biological disturbance that can lead to secondary energy failure and cell death via necrosis and/or apoptosis.

In conclusion, we found that H_2_ gas ventilation combined with TH is associated with higher CBV and lower ScO_2_ after HI insult compared with TH alone and thus we speculate that the CBV increase in the TH + H_2_ group reflects the ability of H_2_ gas to ameliorate the hemodynamic impairment induced by TH. This impact of H_2_ gas on cerebral hemodynamics and oxygen metabolism may provide a key to elucidating its neuroprotective mechanism.

## Methods

### Ethical approval and informed consent

The study protocol was approved by the Kagawa University Animal Care and Use Committee (15070–1) and was conducted in accordance with the Animal Research: Reporting In Vivo Experiments (ARRIVE) guidelines and all other applicable guidelines and regulations.

### Animal procedures

Twenty-eight newborn piglets (Camborough; Daiwa Chikusan, Kagawa, Japan) within 24 h of birth and weighing 1.5–2.1 kg were obtained for the study and divided into three groups: HI-insulted piglets treated with NT (NT group, n = 10), HI-insulted piglets treated with TH (TH group, n = 10), and HI-insulted piglets treated with TH and H_2_ gas ventilation (TH + H_2_ group, n = 8). The piglets in this study included four additional dead piglets that were excluded from a previous study^12^ (NT group, n = 2; TH group, n = 2) and two additional piglets in the TH + H_2_ group.

### Anesthesia, ventilation, and monitoring of physiological variables

The piglets were initially anesthetized with 1%–2% isoflurane in air using a facemask. Each piglet was then intubated and mechanically ventilated using an infant ventilator. The umbilical vein and artery were cannulated using a neonatal umbilical catheter for drip infusion and blood pressure monitoring/blood sampling, respectively. After cannulation, pancuronium bromide was used at an initial dose of 0.1 mg/kg, followed by infusion at 0.1 mg/kg/h to induce paralysis. Next, fentanyl citrate was administered at an initial dose of 10 μg/kg, followed by infusion at 5 μg/kg/h for anesthesia. A maintenance solution of electrolytes plus 2.7% glucose (KN3B; Otsuka Pharmaceutical Co., Tokyo, Japan) was continuously infused at a rate of 4 mL/kg/h via the umbilical vein. Arterial blood samples were taken throughout the experiment at critical time points and when clinically indicated. Each piglet was then placed under a radiant warmer to maintain a mean (standard deviation [SD]) rectal temperature of 39.0 (0.5)°C. The inspired gas was prepared by mixing oxygen and nitrogen (N_2_) gases to obtain the oxygen concentrations required for the experiment. Ventilation was adjusted to maintain arterial oxygen tension (PaO_2_) and arterial carbon dioxide tension within their normal ranges.

### Near-infrared TRS and analysis

We used a portable three-wavelength near-infrared TRS system (TRS-10, 21; Hamamatsu Photonics K.K., Hamamatsu, Japan) and attached a probe to the head of each piglet. The light emitter and detector optodes were positioned in the parietal region at an interoptode distance of 30 mm. The TRS system at our institution uses a time-correlated single-photon counting technique for detection and has been detailed elsewhere^27–29^. The oxyhemoglobin and deoxyhemoglobin concentrations were calculated from their absorption coefficients using equations assuming that background absorption is due only to 85% (by volume) water. ScO_2_ and CBV were calculated as previously described^27–29^.

### Amplitude-integrated electroencephalography

Neural activity was measured by amplitude-integrated electroencephalography (aEEG) (Nicolet One; Cardinal Health, Inc., Dublin, OH). All electrical devices and the copper mesh shield were grounded. The signal was displayed on a semi-logarithmic scale at a low speed (6 cm/h). Measurements were conducted every second. Gold-plated electrode needles were placed at the P3 and P4 positions, which corresponded to the left and right parietal regions of the head. A maximum amplitude < 5 µV was defined as low-amplitude EEG (LAEEG).

### Hypoxic-ischemic insult protocol

Because the details were reported in our previous studies^30,31^, we provide only a brief outline of the HI insult protocol here. Hypoxia was induced by reducing the inspired oxygen concentration of the ventilator to 4% after at least 120 min of stabilization from the initial anesthetic induction. To obtain an LAEEG pattern (< 5 µV), the inspired oxygen concentration was further reduced if necessary, with adjustments as required to avoid cardiopulmonary arrest. From the beginning of the LAEEG, the insult was continued for 30 min. FiO_2_ was decreased (1% decrements) or increased (1% increments) during the insult to maintain the LAEEG, heart rate (HR) (> 130 beats/min), and mean arterial blood pressure (MABP) (> 70% of baseline). LAEEG was maintained for 20 min. For the final 10 min of the 30-min insult, if the MABP exceeded 70% of the baseline, hypotension was induced by decreasing the FiO_2_. Resuscitation was performed when the CBV value dropped below 30% and/or the MABP declined below 70% of baseline. Hypoxia was terminated by resuscitation with 100% oxygen. NaHCO_3_ was used to correct a base deficit (base excess below − 5.0 mEq/L) to maintain a pH of 7.3–7.5. After 10 min of 100% FiO_2_, the ventilator rate and FiO_2_ were gradually reduced to maintain an SpO_2_ of 95–98%. We measured blood gas, glucose, lactate, and hemoglobin levels using a blood gas analyzer (ABL90 FLEX PLUS; Radiometer Co., Ltd., Copenhagen, Denmark).

### Post-insult treatment

After the HI insult, the 28 piglets were randomized into three groups: HI insult with normothermia (NT group, n = 10), HI insult with TH (TH group, 33.5 ± 0.5 °C, n = 10) and HI insult with TH with H_2_ ventilation (TH + H_2_ group, 2.1–2.7% H_2_, n = 8). Whole-body hypothermia was achieved using a cooling blanket (Medicool; MAC8 Inc., Tokyo, Japan) after resuscitation. The piglets were cooled to 33.5 ± 0.5 °C for 24 h and then rewarmed at 1 °C/h using a blanket. Rectal temperature was used as the body temperature. The temperature of the incubator was maintained at 28–32 °C. For H_2_ inhalation, two types of cylinders were used, one containing a gas mixture comprising 3.8% H_2_ and 96.2% N_2_, and the other 100% O_2_, as in a previous study^8^. The H_2_ concentration depended on the oxygen requirement of each piglet. Therefore, the H_2_ concentration was usually between 2.1 and 2.7 (FiO_2_ range, 0.21–0.4) during the therapy. H_2_ gas was delivered through the ventilator for 24 h. The concentration of H_2_ gas was measured by a portable gas monitor (TP-70D; Riken Keiki Co., Ltd., Tokyo, Japan). After 24 h of treatment, the H_2_-N_2_ gas mixture was replaced with an air compressor again. For piglets given TH, their temperature was automatically controlled to maintain the target temperature (rectal temperature, 33–34 °C) during TH and rewarmed at 1 °C/h by a cooling blanket. The anesthesia was stopped at the beginning of the rewarming period. For NT piglets, the rectal temperature was monitored continuously to maintain a normal range (38–39 °C) under the radiant warmer under anesthesia-ventilation for 24 h after the insult. The anesthesia was then stopped and the piglet was extubated.

### Histopathology

On day 5 after the insult, the brain of each animal was perfused with 0.9% saline and 4% phosphate-buffered paraformaldehyde. Coronal blocks of the gray matter, white matter, hippocampus, and cerebellum were embedded in paraffin and cut with a microtome into 4-μm-thick sections. At regular intervals, three sections of each sample were examined. Terminal deoxynucleotidyl transferase-mediated dUTP nick-end labeling (TUNEL) assays were performed with an ApopTag Plus Peroxidase In Situ Apoptosis Detection Kit (ApopTag; EMD Millipore Corp., Burlington, MA) according to the manufacturer’s protocol. TUNEL (+) cells were counted in three areas of the cortical gray matter—the dorsal cortex (DCx), sensorimotor cortex (SMCx), and mid-temporal cortex (MTCx)—as previously reported^12^.

### Data analysis

GraphPad Prism 7.02 (GraphPad Software, La Jolla, CA) was used for all statistical analyses. All values are expressed as the mean ± SD for physiological and blood gas data and for the duration of LAEEG after the insult in the TH and TH + H_2_ groups. Physiological data, blood gas data, total duration of LAEEG, and measurement of HR, MABP, CBV, and ScO_2_ were compared among the three groups at each time point using repeated two-way repeated measures analysis of variance (ANOVA) followed by Tukey’s post hoc analysis. For the comparison of each time point with the baseline value, the correlations between the duration of LAEEG after the insult and the CBV difference after the HI insult were calculated by using Spearman’s analysis. A *p* value < 0.05 was considered significant.

## Data Availability

The datasets generated during and/or analyzed during the present study are available from the corresponding author on reasonable request.

## References

[1] Gluckman PD, Wyatt JS, Azzopardi D, Ballard R, Edwards AD, Ferriero DM (2005). Selective head cooling with mild systemic hypothermia after neonatal encephalopathy: Multicentre randomised trial. Lancet (London, England)..

[2] Jacobs, S.E., Berg, M., Hunt, R., Tarnow‐Mordi, W.O., Inder, T.E., Davis, P.G. Cooling for newborns with hypoxic ischaemic encephalopathy. *Cochrane Database Syst. Rev.* 1 (2013).

[3] Shankaran S, Pappas A, McDonald SA, Vohr BR, Hintz SR, Yolton K (2012). Childhood outcomes after hypothermia for neonatal encephalopathy. N. Engl. J. Med..

[4] Natarajan G, Pappas A, Shankaran S (2016). Outcomes in childhood following therapeutic hypothermia for neonatal hypoxic-ischemic encephalopathy (HIE). Semin. Perinatol..

[5] Ohsawa I, Ishikawa M, Takahashi K, Watanabe M, Nishimaki K, Yamagata K (2007). Hydrogen acts as a therapeutic antioxidant by selectively reducing cytotoxic oxygen radicals. Nat. Med..

[6] Htun, Y., Nakamura, S., Kusaka, T. Hydrogen and therapeutic gases for neonatal hypoxic-ischemic encephalopathy: Potential neuroprotective adjuncts in translational research. *Pediatr. Res.* (2020). 10.1038/s41390-020-0998-z 32505123

[7] Liu L, Xie K, Chen H, Dong X, Li Y, Yu Y (2014). Inhalation of hydrogen gas attenuates brain injury in mice with cecal ligation and puncture via inhibiting neuroinflammation, oxidative stress and neuronal apoptosis. Brain Res..

[8] Htun Y, Nakamura S, Nakao Y, Mitsuie T, Nakamura M, Yamato S (2019). Hydrogen ventilation combined with mild hypothermia improves short-term neurological outcomes in a 5-day neonatal hypoxia-ischaemia piglet model. Sci. Rep..

[9] Nakamura S, Koyano K, Jinnai W, Hamano S, Yasuda S, Konishi Y (2015). Simultaneous measurement of cerebral hemoglobin oxygen saturation and blood volume in asphyxiated neonates by near-infrared time-resolved spectroscopy. Brain Dev..

[10] Jinnai W, Nakamura S, Koyano K, Yamato S, Wakabayashi T, Htun Y (2018). Relationship between prolonged neural suppression and cerebral hemodynamic dysfunction during hypothermia in asphyxiated piglets. Brain Dev..

[11] Buckley EM, Patel SD, Miller BF, Franceschini MA, Vannucci SJ (2015). In vivo monitoring of cerebral hemodynamics in the immature rat: Effects of hypoxia-ischemia and hypothermia. Dev. Neurosci..

[12] Htun Y, Nakamura S, Nakao Y, Mitsuie T, Nakamura M (2019). Hydrogen ventilation combined with mild hypothermia improves short-term neurological outcomes in a 5-day neonatal hypoxia-ischaemia piglet model. Sci. Rep..

[13] Marks KA, Mallard EC, Roberts I, Williams CE, Sirimanne ES, Johnston B (1996). Delayed vasodilation and altered oxygenation after cerebral ischemia in fetal sheep. Pediatr. Res..

[14] Nakamura S, Kusaka T, Koyano K, Miki T, Ueno M, Jinnai W (2014). Relationship between early changes in cerebral blood volume and electrocortical activity after hypoxic-ischemic insult in newborn piglets. Brain Dev..

[15] Nakamura M, Jinnai W, Hamano S, Nakamura S, Koyano K, Chiba Y (2015). Cerebral blood volume measurement using near-infrared time-resolved spectroscopy and histopathological evaluation after hypoxic-ischemic insult in newborn piglets. Int. J. Dev. Neurosci. Off. J. Int. Soc. Dev. Neurosci..

[16] Meek JH, Elwell CE, McCormick DC, Edwards AD, Townsend JP, Stewart AL (1999). Abnormal cerebral haemodynamics in perinatally asphyxiated neonates related to outcome. Arch. Dis. Child. Fetal Neonatal Ed..

[17] Toet MC, Flinterman A, Laar I, Vries JW, Bennink GB, Uiterwaal CS (2005). Cerebral oxygen saturation and electrical brain activity before, during, and up to 36 hours after arterial switch procedure in neonates without pre-existing brain damage: Its relationship to neurodevelopmental outcome. Exp. Brain Res..

[18] Okubo K, Itoh S, Isobe K, Kusaka T, Nagano K, Kondo M (2001). Cerebral metabolism and regional cerebral blood flow during moderate systemic cooling in newborn piglets. Pediatr. Int..

[19] Gunn AJ, Gunn TR, de Haan HH, Williams CE, Gluckman PD (1997). Dramatic neuronal rescue with prolonged selective head cooling after ischemia in fetal lambs. J. Clin. Investig..

[20] Wintermark P, Hansen A, Warfield SK, Dukhovny D, Soul JS (2014). Near-infrared spectroscopy versus magnetic resonance imaging to study brain perfusion in newborns with hypoxic-ischemic encephalopathy treated with hypothermia. Neuroimage.

[21] Wassink G, Gunn ER, Drury PP, Bennet L, Gunn AJ (2014). The mechanisms and treatment of asphyxial encephalopathy. Front. Neurosci..

[22] Domoki F, Olah O, Zimmermann A, Nemeth I, Toth-Szuki V, Hugyecz M (2010). Hydrogen is neuroprotective and preserves cerebrovascular reactivity in asphyxiated newborn pigs. Pediatr. Res..

[23] Nagatani K, Wada K, Takeuchi S, Kobayashi H, Uozumi Y, Otani N (2012). Effect of hydrogen gas on the survival rate of mice following global cerebral ischemia. Shock.

[24] Fellman V, Raivio KO (1997). Reperfusion injury as the mechanism of brain damage after perinatal asphyxia. Pediatr. Res..

[25] Chen CH, Manaenko A, Zhan Y, Liu WW, Ostrowki RP, Tang J (2010). Hydrogen gas reduced acute hyperglycemia-enhanced hemorrhagic transformation in a focal ischemia rat model. Neuroscience.

[26] Hayashida K, Sano M, Ohsawa I, Shinmura K, Tamaki K, Kimura K (2008). Inhalation of hydrogen gas reduces infarct size in the rat model of myocardial ischemia-reperfusion injury. Biochem. Biophys. Res. Commun..

[27] Ijichi S, Kusaka T, Isobe K, Islam F, Okubo K, Okada H (2005). Quantification of cerebral hemoglobin as a function of oxygenation using near-infrared time-resolved spectroscopy in a piglet model of hypoxia. J. Biomed. Opt..

[28] Ijichi S, Kusaka T, Isobe K, Okubo K, Kawada K, Namba M (2005). Developmental changes of optical properties in neonates determined by near-infrared time-resolved spectroscopy. Pediatr. Res..

[29] Kusaka T, Isobe K, Yasuda S, Koyano K, Nakamura S, Nakamura M (2014). Evaluation of cerebral circulation and oxygen metabolism in infants using near-infrared light. Brain Dev..

[30] Nakamura S, Kusaka T, Yasuda S, Ueno M, Miki T, Koyano K (2013). Cerebral blood volume combined with amplitude-integrated EEG can be a suitable guide to control hypoxic/ischemic insult in a piglet model. Brain Dev..

[31] Yamato SH, Nakamura S, Htun Y, Nakamura M, Jinnai W, Nakao Y (2020). Intravenous edaravone plus therapeutic hypothermia offers limited neuroprotection in the hypoxic-ischaemic newborn piglet. Neonatology.

